# *In Silico* Analysis of Potential Vaccine Antigens for the Treatment of Crimean-Congo Hemorrhagic Fever Virus (Cchfv)

**DOI:** 10.1007/s11095-025-04002-2

**Published:** 2026-01-15

**Authors:** Deniz Tülümen, Esra Aydemir, Furkan Ayaz

**Affiliations:** Department of Molecular Biology and Genetics, Faculty of Engineering and Natural Sciences, Istinye University, Istanbul, 34010 Türkiye

**Keywords:** crimean-congo hemorrhagic fever virus (CCHFV), *hyalomma marginatum*, *in-silico*, vaccine candidates

## Abstract

**Background:**

Crimean-Congo hemorrhagic fever virus (CCHFV), identified by the World Health Organization(WHO) as a potential epidemic threat, is a tick-borne Nairovirus primarily transmitted by Hyalomma marginatum species. Since its first detection in the 1940s, CCHFV has spread to multiple regions worldwide and remains a major public health concern due to its high fatality rate and expanding geographic distribution. The virus can be transmitted through tick bites or contact with infected individuals, and no licensed vaccine is currently available to prevent infection.

**Methods:**

In this study, two CCHFV proteins, Q8JSZ3 (GP_CCHFI) and Q6TQR6 (L_CCHFI), were retrieved from public databases and analyzed using bioinformatic tools to explore their potential as vaccine candidates.

**Results:**

The computational analyses revealed that both proteins possess non-toxic characteristics and show promise for future vaccine design.

**Conclusions:**

These findings provide a preliminary in-silico framework that may guide the development of effective vaccines against CCHFV.

## Introduction

Crimean-Congo hemorrhagic fever virus (CCHFV) is a tick-borne viral disease with a wide geographic distribution that frequently affects humans. It belongs to the *Nairovirus* genus within the *Bunyaviridae* family and is classified as an arbovirus because it is transmitted by arthropods, primarily ticks [1]. Ticks serve as both vectors and reservoirs for CCHFV, with *Hyalomma marginatum* species being the primary source of human infections [2].The first recorded outbreak of CCHFV occurred in the 1940 s among Russian soldiers in the Republic of Crimea, causing acute febrile illness, and has since spread across several regions of the world [3]. According to the World Health Organization (WHO), CCHFV is now endemic in more than 30 countries across Africa, Asia, Eastern Europe, and the Middle East, posing a major global public health concern https://www.who.int/news-room/fact-sheets/detail/crimean-congo-haemorrhagic-fever. Globally, thousands of human cases are reported each year, with case fatality rates ranging from 5 to 30%, depending on healthcare access and outbreak conditions https://www.cdc.gov/crimean-congo-hemorrhagic/about/?CDC_AAref_Val=https://www.cdc.gov/vhf/crimean-congo. These numbers highlight the urgent need for new and effective therapeutic and preventive approaches against this highly pathogenic virus.CCHFV can be transmitted through tick bites, direct contact with infected animal blood or tissues, and occasionally through human-to-human transmission via contact with infected patients’ blood or bodily fluids [4]. Structurally, CCHFV is an enveloped, negative-sense, single-stranded RNA virus with a tripartite genome exhibiting significant genetic diversity [5]. In humans, infection can lead to severe viral hemorrhagic fever with a mortality rate reaching up to 30% [6]. Early symptoms typically include high fever, nausea, abdominal pain, and rash, while later stages are characterized by bleeding disorders due to coagulation abnormalities [7].

Currently, there is no licensed vaccine or specific antiviral treatment for CCHFV infection. Ribavirin has been used as an antiviral therapy but has shown limited efficacy [8]. Due to its high fatality rate, mutation potential, and transmission risk, CCHFV represents a serious public health concern and has been designated as a priority pathogen for research by the World Health Organization (WHO) https://www.who.int/activities/prioritizing-diseases-for-research-and-development-in-emergency-contexts. Therefore, new strategies involving computational and artificial intelligence-based approaches are essential for developing effective therapeutics and vaccines against this virus.

In this context, two key proteins specific to CCHFV were selected from the UniProt database for comprehensive *in silico* characterization https://www.uniprot.org/. The envelope glycoprotein precursor (GP_CCHFV; UniProt ID: Q8JSZ3) undergoes post-translational cleavage into mature glycoproteins Gn and Gc, which are embedded in the viral envelope and mediate critical steps of viral infection. Gn facilitates the initial attachment of the virion to host cell receptors, while Gc drives the membrane fusion process required for viral entry https://www.uniprot.org/uniprotkb/Q8JSZ3/entry. Both glycoproteins play crucial roles in immune evasion by shielding conserved epitopes and modulating host immune responses. Owing to their surface exposure and involvement in host–virus interactions, these proteins are primary targets for neutralizing antibodies and have been recognized as promising candidates for vaccine design.

The RNA-dependent RNA polymerase (L_CCHFV; UniProt ID: Q6TQR6) is a large multifunctional enzyme responsible for the replication and transcription of the viral genome https://www.uniprot.org/uniprotkb/Q6TQR6/entry. This protein contains several conserved motifs that are characteristic of viral polymerases, including domains for RNA synthesis, capping, and proofreading. The L protein also exhibits deubiquitinating and deISGylating activities that help the virus counteract the host’s antiviral defense mechanisms. Due to its essential role in viral replication and its high degree of sequence conservation across CCHFV strains, the L protein represents a potential broad-spectrum antiviral and immunogenic target.

**Fig. 1:**
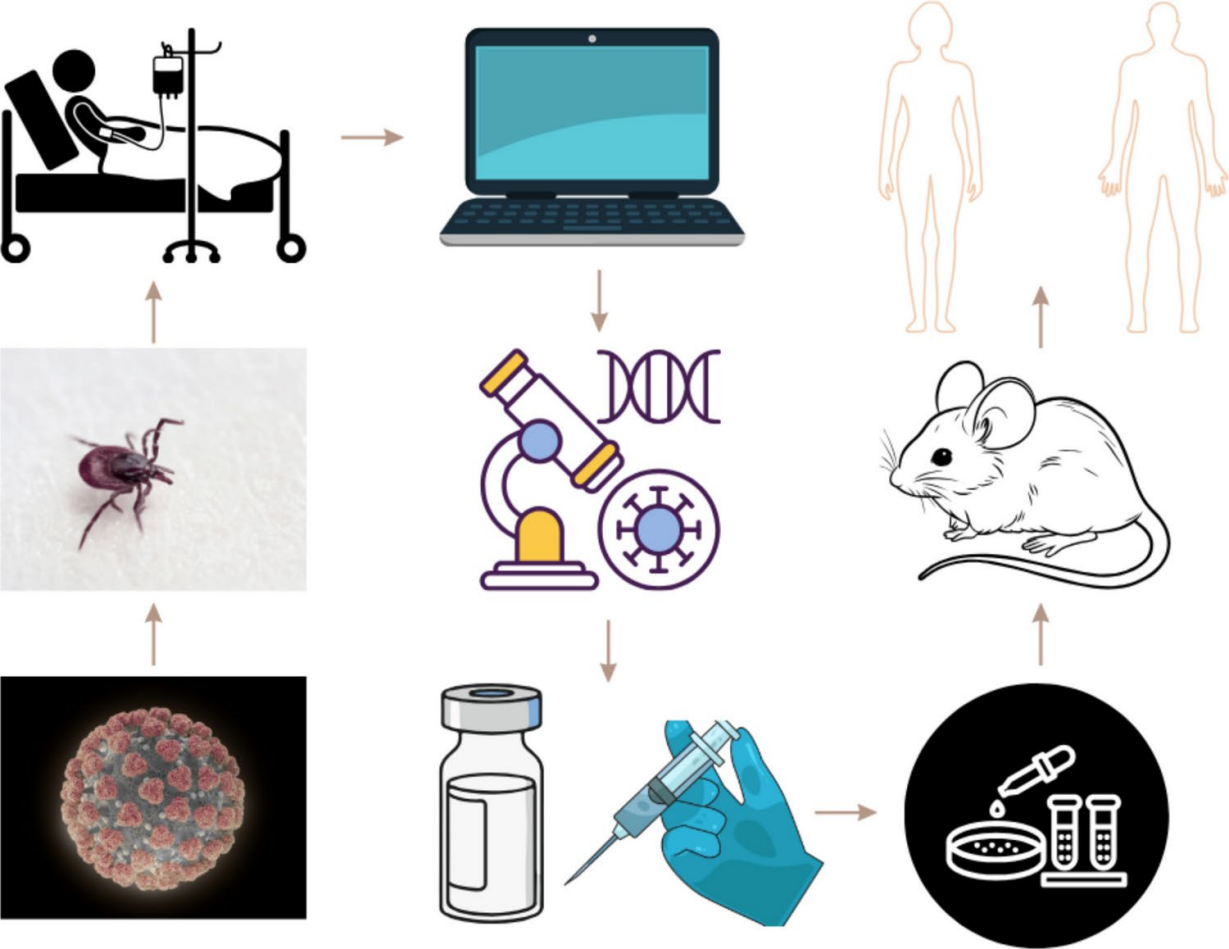
Translational workflow for CCHFV vaccine development: from virus and natural transmission to *in silico* design, *in vitro* validation, and preclinical and clinical evaluation: Schematic representation of the Crimean-Congo hemorrhagic fever virus (CCHFV) transmission and vaccine development process. The virus is transmitted via ticks to humans (patients). Target proteins are identified and analyzed through *in silico* modeling, followed by *in vitro* cellular studies for validation. Candidate vaccines are then evaluated in preclinical mouse models before progressing to human trials.

In this study, comprehensive *in-silico* analyses were performed to characterize the physicochemical, structural, antigenic, allergenic, and toxic properties of these two proteins. Given the high pathogenicity and mutation rate of CCHFV, the results may provide valuable insights for the rational design of future vaccine candidates (Fig. 1).

## Material and methods

### Physicochemical properties of proteins

The physicochemical characteristics of the selected CCHFV proteins (Q8JSZ3 and Q6TQR6) were analyzed using the ExPASy ProtParam server [9]. The amino acid sequences in FASTA format were submitted as input, and parameters such as molecular weight, theoretical isoelectric point (pI), amino acid composition, instability index, aliphatic index, and GRAVY (Grand Average of Hydropathicity) values were calculated. These parameters were used to predict the stability, solubility, and hydrophobic nature of the proteins under physiological conditions.

### Estimation of secondary structures of proteins

The GOR IV algorithm available through the Prabi database was used to predict the secondary structures of the proteins from their amino acid sequences [10]. This tool estimates the proportion of alpha helices, beta strands, and random coils based on information theory and Bayesian statistics. Default parameters were applied to obtain the secondary structure composition of each protein.

### Predicting the subcellular localization of proteins

To determine the likely cellular localization of each viral protein, DeepLoc-Pro v1.0 was employed https://services.healthtech.dtu.dk/services/DeepLocPro-1.0/. FASTA sequences were used as input, and predictions were made using default thresholds. Determining localization helps infer the biological role of each protein and assess their accessibility to host immune responses, which is critical for vaccine design.

### Estimation of solubility of proteins in Escherichia coli

The solubility of each protein upon overexpression in *Escherichia coli* was estimated using SOLUPROT v1.0 https://loschmidt.chemi.muni.cz/soluprot/.The analysis was conducted using the default solubility cutoff value of 0.45. *E. coli* was selected as a model organism because it is a widely used host system for recombinant protein expression in experimental validation studies [11].

### Estimation of antigenic values ​​of proteins

Antigenicity of the selected proteins was evaluated using VaxiJen v2.0 [12]. The sequences were analyzed with a threshold of 0.4, as recommended for viral proteins. Proteins with scores above this value were classified as probable antigens. This analysis aimed to identify proteins capable of eliciting immune responses, providing initial insights for in-silico vaccine design.

### Estimation of allergenicity of proteins

Allergenicity was assessed using the AlgPred server [13]. MEME/MAST motif searches and IgE epitope mapping were performed to identify potential allergenic motifs within the protein sequences. Proteins predicted as non-allergenic were prioritized since vaccine candidates must not trigger allergic responses in the host.

### Toxicity prediction of proteins

The CSM-Toxin server was used to evaluate the toxicity of the selected proteins [14]. This structure-independent classifier uses graph-based signatures derived from amino acid sequences to identify toxic peptides. Only non-toxic proteins were considered suitable for further evaluation as vaccine candidates.

### Transmembrane helix prediction of proteins

Transmembrane helix prediction of the selected proteins was performed using the DeepTMHMM v1.0 server https://services.healthtech.dtu.dk/services/DeepTMHMM-1.0/. DeepTMHMM is a deep learning–based protein topology prediction tool that identifies α-helices and β-barrel regions within transmembrane proteins and provides both graphical and textual outputs. This analysis is crucial for vaccine design, as transmembrane proteins account for approximately 50–60% of current vaccine and drug targets https://www.biorxiv.org/content/10.1101/2022.04.08.487609v1.

### Protein 3D modeling

The 3D structures of the selected proteins, Q8JSZ3 and Q6TQR6, were predicted using the AlphaFold Server Beta https://alphafoldserver.com/. Protein chain information and identifiers were retrieved from the UniProt database and visualized in PyMOL https://www.pymol.org/.

## Results

### Physicochemical properties of target proteins

The physicochemical analyses of the selected target proteins were performed using the ExPASy ProtParam database. According to the results, protein **Q8JSZ3** consists of 1684 amino acids with a molecular weight of approximately 186.61 kDa. The theoretical isoelectric point (pI) was determined to be 7.40, indicating the approximate pH at which the protein carries no net charge and would remain stationary during two-dimensional gel electrophoresis.

The distribution of charged residues at neutral pH provides insights into the protein’s localization. The total number of negatively charged residues (Asp + Glu) was determined to be 176 for this protein. Proteins localized within the cell generally exhibit higher numbers of negatively charged residues than extracellular proteins. The total number of positively charged residues (Arg + Lys) was found to be 177 for the Q8JSZ3 protein.

The extinction coefficient represents the amount of light absorbed by the protein at a specific wavelength and is essential for determining protein concentration and purification using a spectrophotometer. This coefficient can be estimated based on the amino acid composition of the protein [15]. When measured in water at 280 nm, the absorbance value of the protein at 1% concentration was 0.930, and when all Cys residues were paired as cystine, the extinction coefficient was 173,445. At 0.1% concentration, when all Cys residues were reduced, the extinction coefficient was found to be 168,570.

The estimated half-life of a protein refers to the time required for half of the protein molecules to be degraded in the cell after synthesis. This time is estimated according to the “N-end rule”, which applies to model organisms such as humans, yeast, and E. coli [16]. This rule is particularly important in *in vivo* experiments to evaluate protein stability [17]. Based on this rule, the half-life times of the Q8JSZ3 protein in human, yeast, and *E. coli* were calculated (Table I).

The instability index of a protein is used to predict its stability. A value above 40 indicates that the protein is unstable, while a value below 40 indicates stability. However, unstable proteins may contain certain dipeptides not present in stable proteins [18]. The instability index of the Q8JSZ3 protein was found to be 45.85, which exceeds 40, and therefore the protein is considered unstable.

The aliphatic index represents the relative volume occupied by aliphatic side chains (alanine, valine, isoleucine, and leucine) and is a positive factor contributing to the thermostability of globular proteins [19]. The aliphatic index of the Q8JSZ3 protein was calculated as 84.14.

The grand average of hydropathy (GRAVY) value was obtained by dividing the sum of hydropathic indices of all amino acids by the number of residues in the sequence [20]. This value indicates whether the protein is predominantly hydrophobic or hydrophilic. Higher values correspond to more hydrophobic proteins, which tend to be buried within the protein core, while lower (negative) values indicate hydrophilic regions, generally located on the protein surface. According to this, the GRAVY value of the Q8JSZ3 protein was found to be −0.183, indicating that the protein is more hydrophilic due to its negative value (Table I).

The amino acid composition of the Q8JSZ3 protein, showing the percentage of each amino acid residue, is shown (Table II).

**Table I:** Physicochemical Analyses of Q8JSZ3 Protein

Number of amino acids	1684
Molecular weight	186,589.20
Theoretical pI	7.40
Asp + Glu(Total number of negatively charged residues)	176
Arg + Lys(Total number of positively residues)	177
Extinction coefficients	Ext. coefficient 173,445 Abs 0.1% (= 1 g/l) 0.930, assuming all pairs of Cys residues form cystines Ext. coefficient 168,570 Abs 0.1% (= 1 g/l) 0.903, assuming all Cys residues are reduced
Estimated half-life	30 h (mammalian reticulocytes, *in vitro*) > 20 h (yeast, *in vivo*) > 10 h (Escherichia coli, *in vivo*)
Instability index	The instability index is computed to be 45.85 This classifies the protein as unstable
Aliphatic index	84.14
Grand average of hydropathicity (GRAVY)	−0.183

**Table II:** Q8JSZ3 Protein in Amino Acid Composition

Amino acid composition:	
Ala (A) 69	4.1%
Arg (R) 74	4.4%
Asn (N) 64	3.8%
Asp (D) 71	4.2%
Cys (C) 79	4.7%
Gln (Q) 45	2.7%
Glu (E) 105	6.2%
Gly (G) 114	6.8%
His (H) 51	3.0%
Ile (I) 108	6.4%
Leu (L) 164	9.7%
Lys (K) 103	6.1%
Met (M) 29	1.7%
Phe (F) 60	3.6%
Pro (P) 86	5.1%
Ser (S) 150	8.9%
Thr (T) 151	9.0%
Trp (W) 19	1.1%
Tyr (Y) 43	2.6%
Val (V) 99	5.9%
Pyl (O) 0 0	0.0%
Sec (U) 0 0	0.0%
(B) 0 0	0.0%
(Z) 0 0	0.0%
(X) 0 0	0.0%

The physicochemical properties of protein **Q6TQR6**, which consists of 3945 amino acids, revealed a molecular weight of approximately 448 kDa. The theoretical isoelectric point (pI) was calculated as 7.54. The total number of negatively charged residues (Asp + Glu) was 497, while the total number of positively charged residues (Arg + Lys) was 500.

The extinction coefficient at 280 nm in water was determined to be 355,645 when all cysteine residues were assumed to form cystine pairs, and 349,520 when all cysteine residues were reduced. At a 1% concentration, the absorbance values were 0.794 and 0.780, respectively.

The estimated half-life, calculated from the N-terminal methionine residue for model organisms (human, yeast, and *E. coli*), is presented. The instability index of the protein was 43.70, which is greater than 40, indicating that the protein is unstable. The aliphatic index was found to be 92.91. Additionally, the GRAVY value of −0.279 indicates that the protein is relatively hydrophilic (Table III). The amino acid composition (in percentage) is summarized (Table IV).

**Table III:** Physicochemical Analyses of Q6TQR6 Protein

Number of amino acids	3945
Molecular weight	447,940.43
Theoretical pI	7.54
Asp + Glu(Total number of negatively charged residues)	497
Arg + Lys(Total number of positively residues)	500
Total number of atoms	63,226
Extinction coefficients	Ext. coefficient 355,645 Abs 0.1% (= 1 g/l) 0.794, assuming all pairs of Cys residues form cystines Ext. coefficient 349,520 Abs 0.1% (= 1 g/l) 0.780, assuming all Cys residues are reduced
Estimated half-life	30 h (mammalian reticulocytes, *in vitro*) > 20 h (yeast, *in vivo*) > 10 h (Escherichia coli, *in vivo*)
Instability index	The instability index is computed to be 43.70 This classifies the protein as unstable
Aliphatic index	92.91
Grand average of hydropathicity (GRAVY)	−0.279

**Table IV:** Amino Acid Composition of Q6TQR6 Protein

Amino acid composition:	
Ala (A) 170	4.3%
Arg (R) 203	5.1%
Asn (N) 206	5.2%
Asp (D) 216	5.5%
Cys (C) 98	2.5%
Gln (Q) 146	3.7%
Glu (E) 281	7.1%
Gly (G) 174	4.4%
His (H) 82	2.1%
Ile (I) 232	5.9%
Leu (L) 485	12.3%
Lys (K) 297	7.5%
Met (M) 88	2.2%
Phe (F) 166	4.2%
Pro (P) 113	2.9%
Ser (S) 378	9.6%
Thr (T) 234	5.9%
Trp (W) 37 0	0.9%
Tyr (Y) 98	2.5%
Val (V) 241	6.1%
Pyl (O) 0 0	0.0%
Sec (U) 0 0	0.0%
(B) 0 0	0.0%
(Z) 0 0	0.0%
(X) 0 0	0.0%

### Secondary Structure Prediction of Target Proteins

**Fig. 2:**
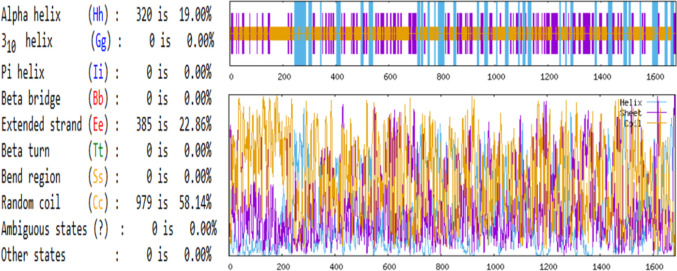
Secondary structure values and schematization of Q8JSZ3 protein predicted by the GOR IV algorithm, indicating 19.00% α-helices, 22.86% extended β-strands, and 58.14% random coils.

The secondary structures of the protein Q8JSZ3 were predicted using the GOR IV algorithm. The proportions of α-helices, extended β-strands, and random coils were found to be 19.00%, 22.86%, and 58.14%, respectively (Fig. 2). The predominance of random coil regions suggests a flexible conformation that may influence its surface exposure and potential immunogenicity.

**Fig. 3:**
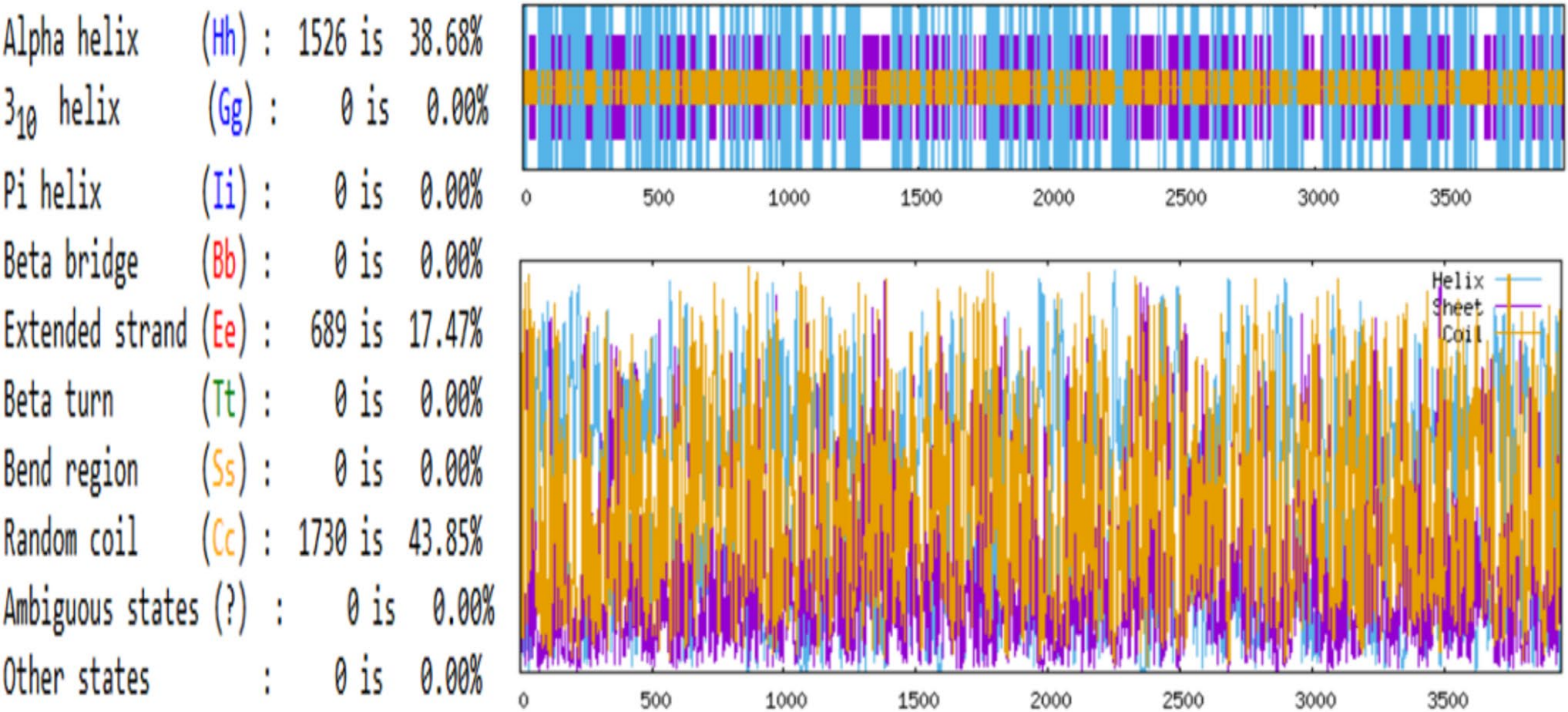
Secondary structure values and schematization of Q6TQR6 protein predicted by the GOR IV algorithm, indicating 38.68% α-helices, 17.47% extended β-strands, and 43.85% random coils.

In the case of **Q6TQR6,** the secondary structure composition consisted of 38.68% α-helices, 17.47% extended β-strands, and 43.85% random coils (Fig. 3).

### Subcellular Localization Prediction of Target Proteins

**Fig. 4:**
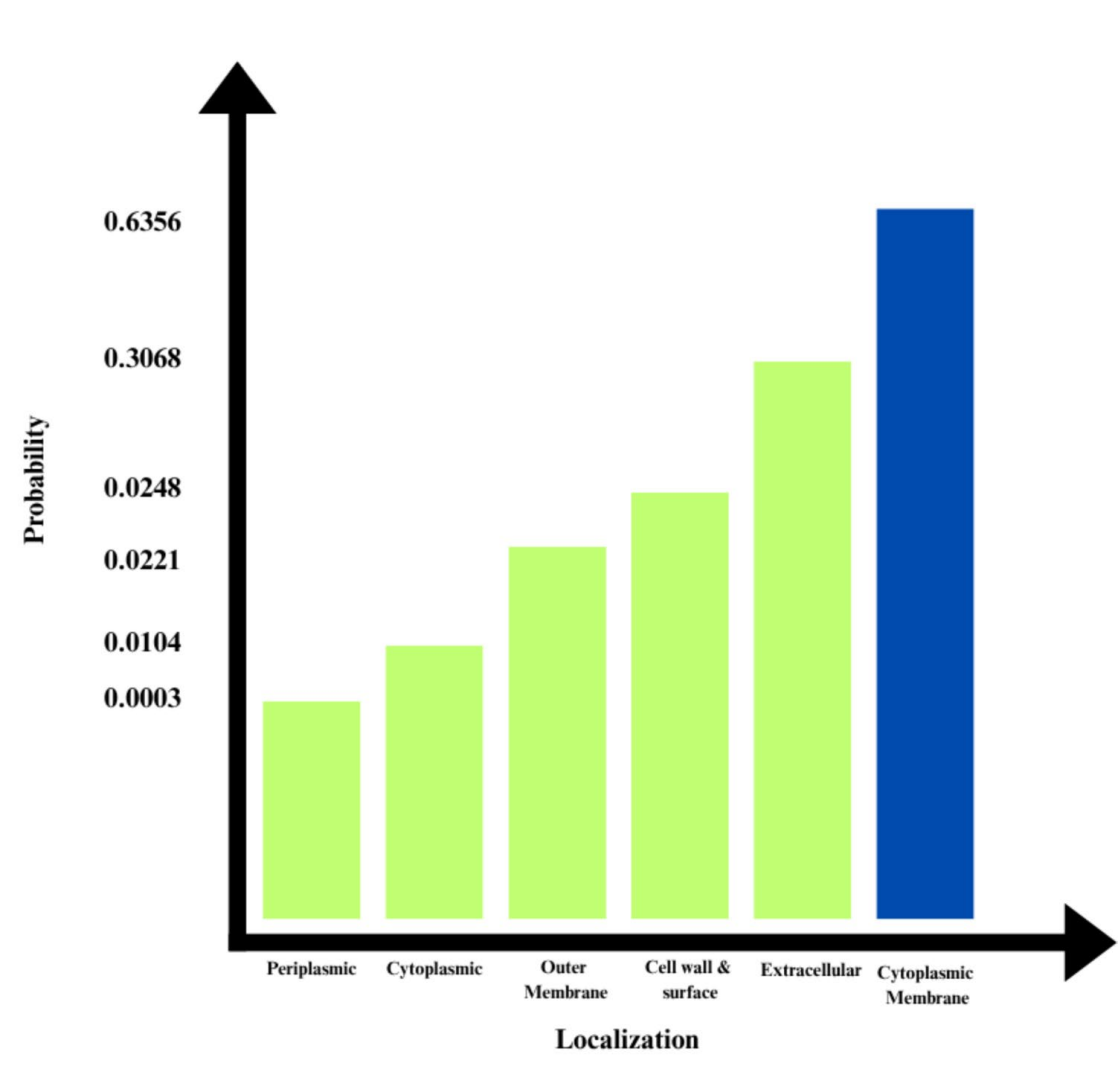
Subcellular localization prediction of the Q8JSZ3 protein obtained using the DeepLoc-Pro 1.0 server: The highest probability was observed for cytoplasmic membrane localization (0.6356), followed by extracellular and outer membrane regions, suggesting a predominant association with the cytoplasmic membrane.

The subcellular localization of the Q8JSZ3 protein was predicted using the DeepLoc-Pro 1.0 server. The analysis revealed that the protein is most likely associated with the cytoplasmic membrane, showing the highest probability score of 0.6356. Lower probabilities were observed for extracellular, outer membrane, and cytoplasmic localizations, indicating a strong membrane-associated profile (Fig. 4).

**Fig. 5:**
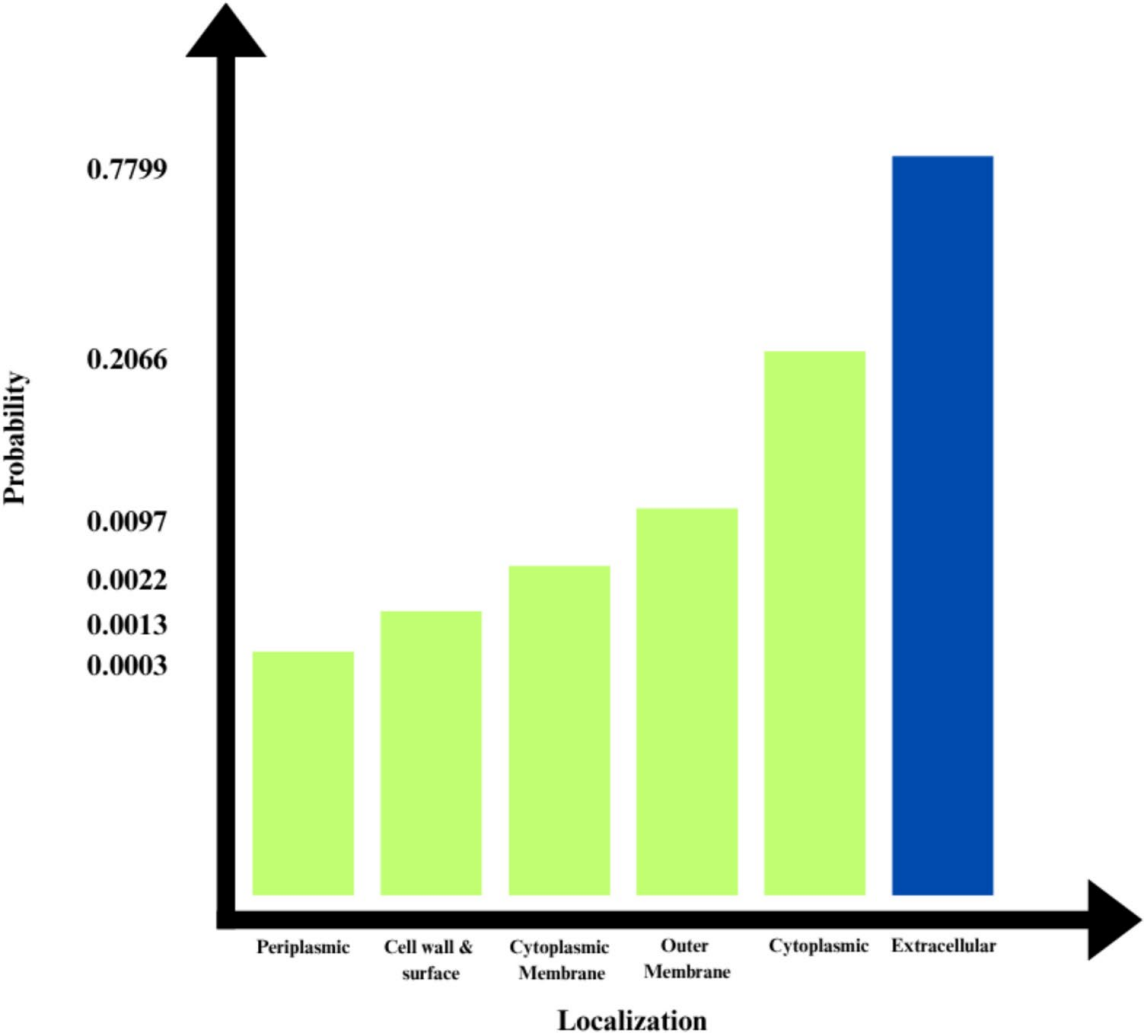
Subcellular localization prediction of the Q6TQR6 protein obtained using the DeepLoc-Pro 1.0 server: The highest probability (0.7799) corresponds to extracellular localization, followed by cytoplasmic and membrane-associated regions, indicating a predominant extracellular distribution.

The subcellular localization of the Q6TQR6 protein was predicted using the DeepLoc-Pro 1.0 server. The protein was primarily localized in the extracellular region with the highest probability value of 0.7799, suggesting that it may play a significant role in extracellular interactions during viral infection (Fig. 5).

### Prediction of Solubility of Target Proteins in Escherichia coli

The solubility of the target proteins in *Escherichia coli* was evaluated using the SOLUPROT v1.0 server. Q8JSZ3 exhibited a solubility value of 0.730, and Q6TQR6 showed a value of 0.729. Both proteins were considered soluble, as their values exceeded the 0.5 threshold.

### Antigenicity Prediction of Selected Proteins

The antigenicity of the selected proteins was predicted using the VaxiJen v2.0 server, which evaluates the probability of a protein being an antigen based on its physicochemical properties rather than sequence alignment [12]. A default threshold of 0.5 was used, where values above this cutoff indicate probable antigenicity. Q8JSZ3 yielded an antigenicity score of 0.5145, and Q6TQR6 scored 0.5020. Both scores exceeded the threshold, classifying the proteins as probable antigens. Although the values were only slightly above the cutoff, they still indicate potential immunogenicity, suggesting that these proteins could serve as promising *in-silico* vaccine targets for CCHFV.

### Allergenicity Prediction of Selected Proteins

The allergenicity of the selected proteins was assessed using the AlgPred database, which predicts allergenic potential based on IgE epitope mapping and motif analysis. Q8JSZ3 and Q6TQR6 were both classified as non-allergenic, and neither protein was predicted to contain any IgE epitopes. This suggests that these proteins are unlikely to elicit IgE-mediated allergic responses, supporting their suitability as potential vaccine targets.

### Toxicity Prediction of Selected Proteins

The toxicity of the target proteins was evaluated using the CSM-Toxin server, which classifies proteins as toxic or non-toxic based on sequence-derived probability scores. Q8JSZ3 and Q6TQR6 were predicted to be non-toxic, indicating that they may be considered as potential vaccine candidates. However, as these predictions are computational, further experimental validation is required to confirm their safety and suitability for vaccine development. Toxicity assessment remains critical for identifying immunogenic proteins with potential clinical applicability.

### Transmembrane Helix Prediction of Target Proteins

**Fig. 6:**
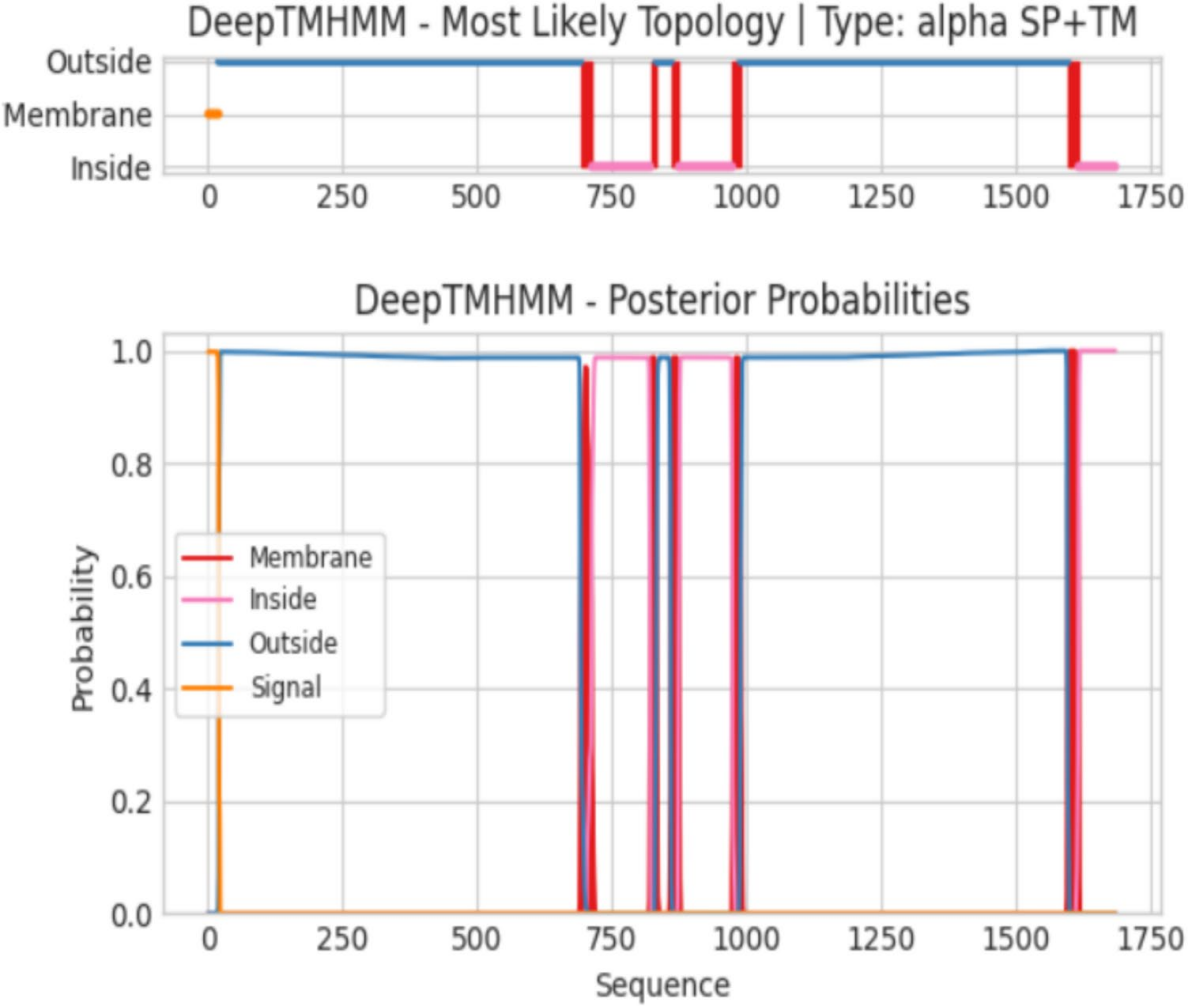
Transmembrane helix prediction of the Q8JSZ3 protein: The red regions represent predicted transmembrane helices, whereas the pink and blue areas correspond to the intracellular and extracellular regions, respectively. A total of five transmembrane helices were identified for this protein.

**Fig. 7:**
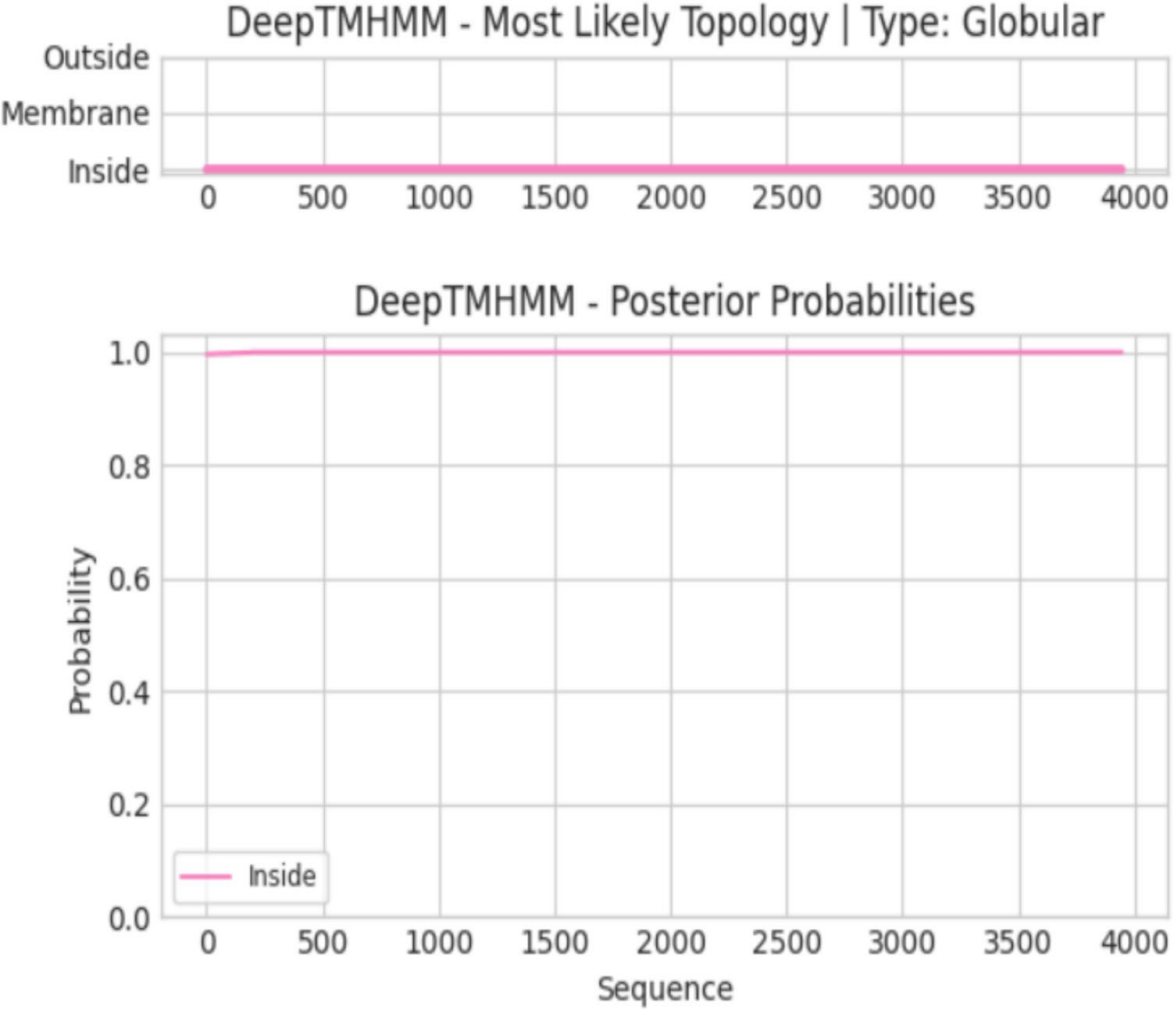
Transmembrane helix prediction of the Q6TQR6 (L_CCHFI) protein: The red regions represent predicted transmembrane helices, whereas the pink and blue areas correspond to the intracellular and extracellular regions, respectively. No transmembrane helices were identified for this protein.

Transmembrane helix prediction was performed using the DeepTMHMM-1.0 database for the selected target proteins. Q8JSZ3 is predicted to contain five transmembrane helices (Fig. 6), whereas Q6TQR6 lacks transmembrane regions (Fig. 7).

### 3D Modeling of Target Proteins

**Fig. 8:**
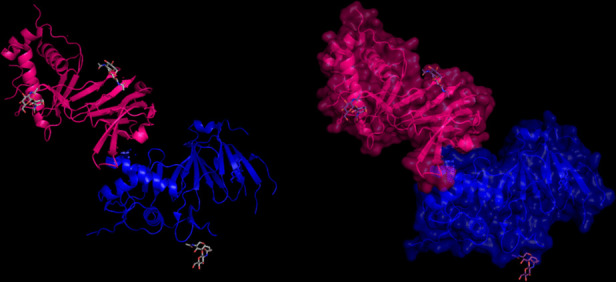
Chain (**a**) (hot pink) and chain (**b**) (blue) of the 6VKF structure were visualized, corresponding to residues 248–515.

**Fig. 9:**
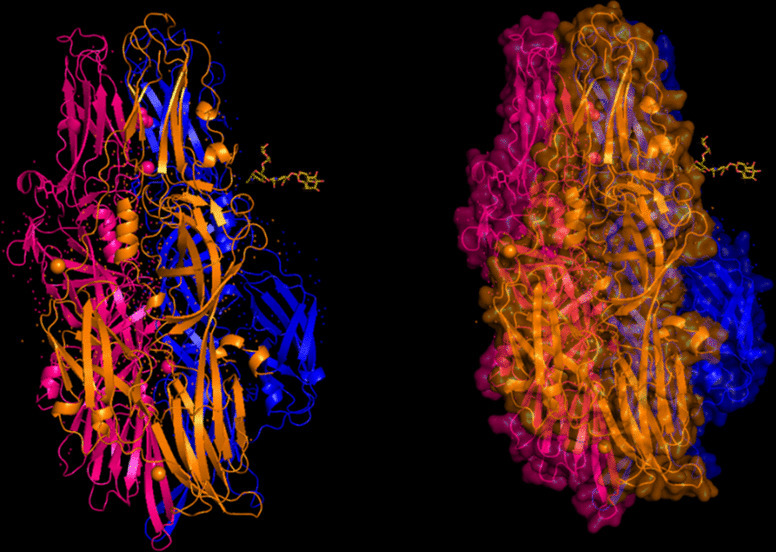
Chains (**a**) (hot pink), (**b**) (blue), and (**c**) (orange) of the 7A59 structure were visualized, corresponding to residues 1041–1561.

**Fig. 10:**
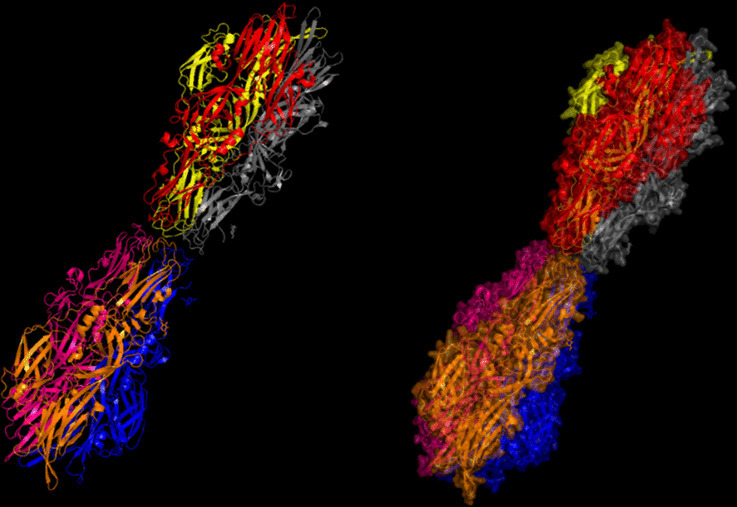
Chains (**a**) (hot pink), (**b**) (blue), (**c**) (orange), (**d**) (yellow), (**e**) (red), and (**f**) (grey) of the 7A5A structure were visualized, corresponding to residues 1041–1572.

**Fig. 11:**
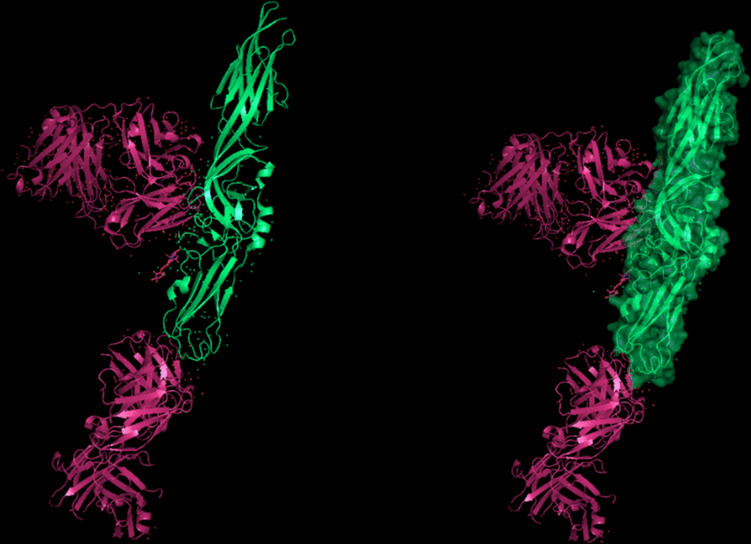
Chain G (limegreen) of the 7L7R structure was visualized, corresponding to residues 1041–1579.

**Fig. 12:**
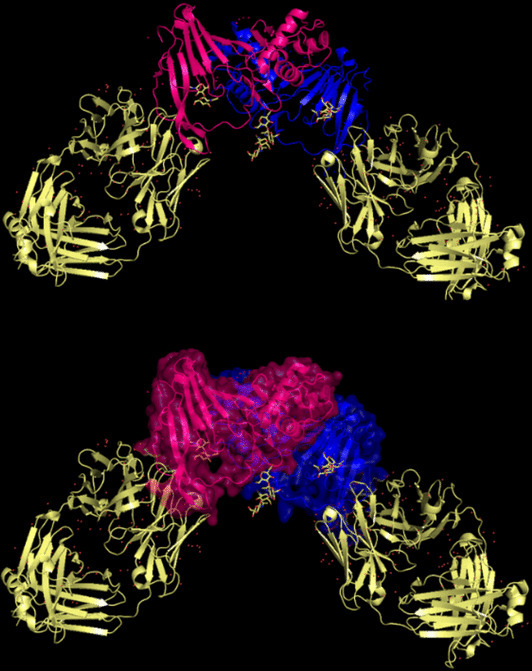
Chains (**a**) (hot pink) and (**b**) (blue) of the 8VVK structure were visualized, corresponding to residues 248–515.

**Fig. 13:**
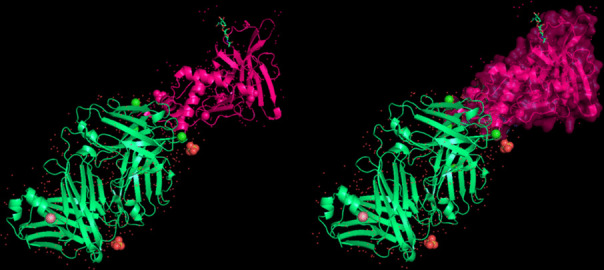
Chain (**a**) (hot pink) of the 8VVL structure was visualized, corresponding to residues 248–515.

The modeling of the proteins was examined. The 3D structure of Q8JSZ3 was downloaded from the UniProt database and visualized in PyMOL, where different chains and regions were colored to highlight structural features (Figs. 8, 9, 10, 11, 12 and 13).

**Fig. 14:**
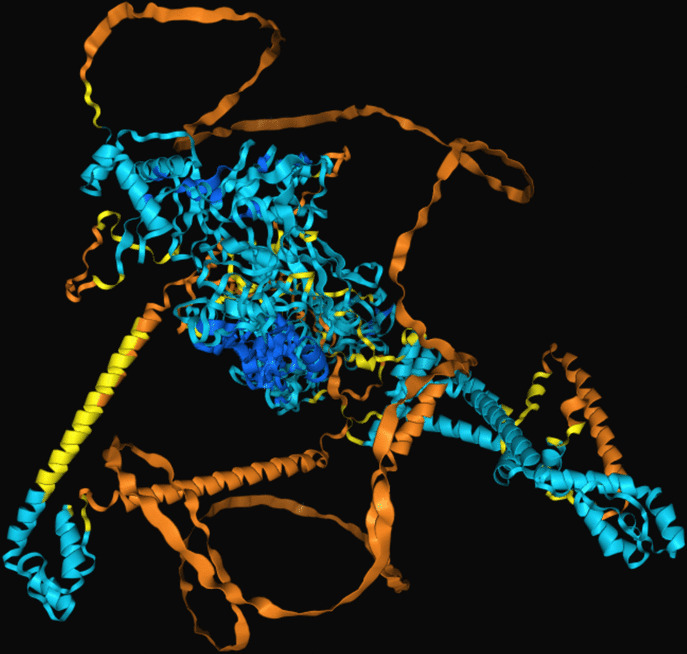
The most reliable 3D model of the Q8JSZ3 protein obtained from AlphaFold DB.

The closest 3D model of the Q8JSZ3 protein was obtained from the AlphaFold DB (Fig. 14). The ipTM–pTM score was 0.6, which is higher than the 0.5 threshold, indicating that the predicted structure is likely to be close to the native conformation [21]

The modeling of the Q6TQR6 protein was examined. The 3D structure of Q6TQR6 was obtained from the UniProt database and visualized in PyMOL, where distinct chains and regions were colored to emphasize structural organization (Figs. 15, 16, 17, 18, 19, 20, 21, 22, 23, 24 and 25).

**Fig. 15:**
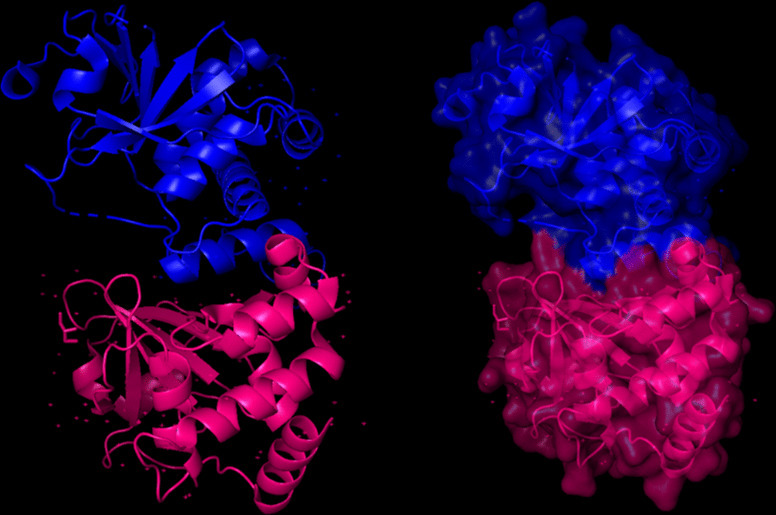
Chains (**a**) (hot pink) and (**b**) (blue) of the 3PHU structure were visualized, corresponding to residues 1–217.

**Fig. 16:**
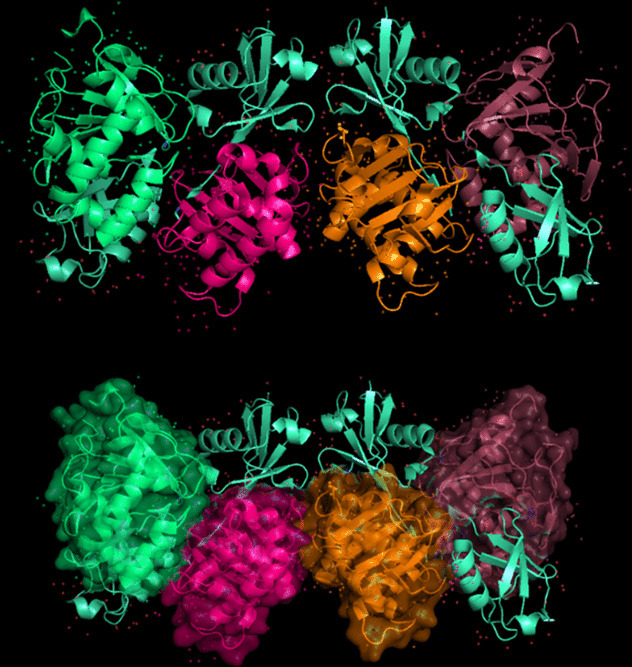
Chains (**a**) (hot pink), (**c**) (orange), (**e**) (red), and (**g**) (lime green) of the 3PHW structure were visualized, corresponding to residues 1–183.

**Fig. 17:**
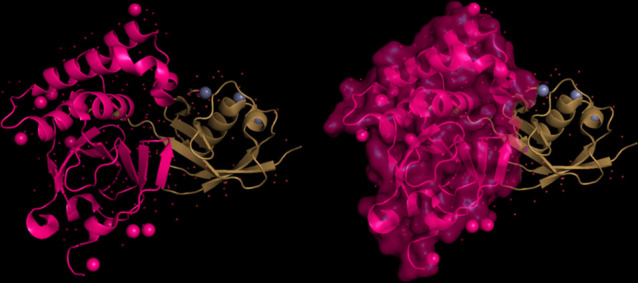
Chain (**a**) (hot pink) of the 3PHX structure was visualized, corresponding to residues 1–183.

**Fig. 18:**
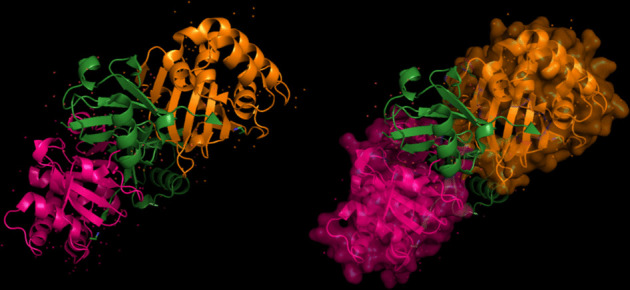
Chains (**a**) (hot pink) and (**c**) (orange) of the 3PRM structure were visualized, corresponding to residues 1–170.

**Fig. 19:**
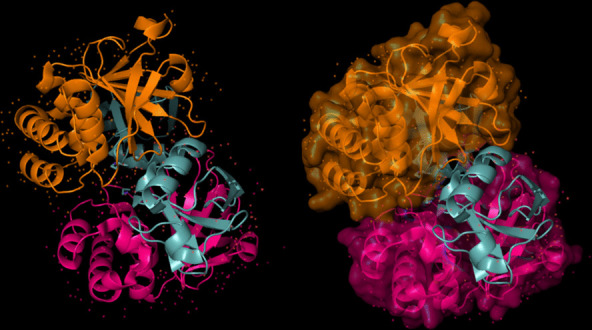
Chains (**a**) (hot pink) and (**c**) (orange) of the 3PRP structure were visualized, corresponding to residues 1–170.

**Fig. 20:**
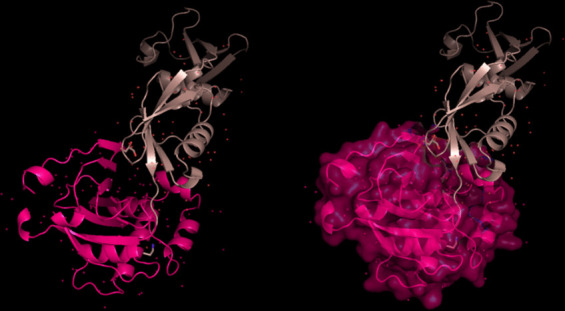
Chain (**a**) (hot pink) of the 3PSE structure was visualized, corresponding to residues 1–169.

**Fig. 21:**
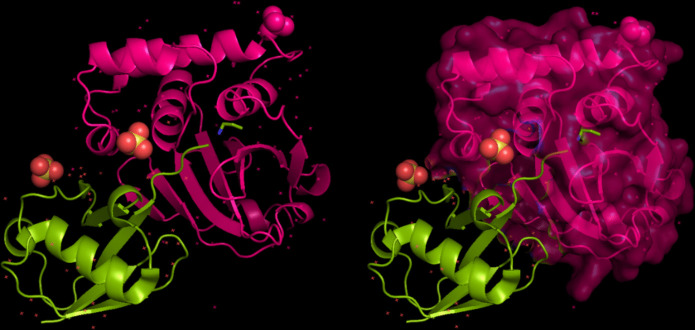
Chain (**a**) (hot pink) of the 3PT2 structure was visualized, corresponding to residues 1–184.

**Fig. 22:**
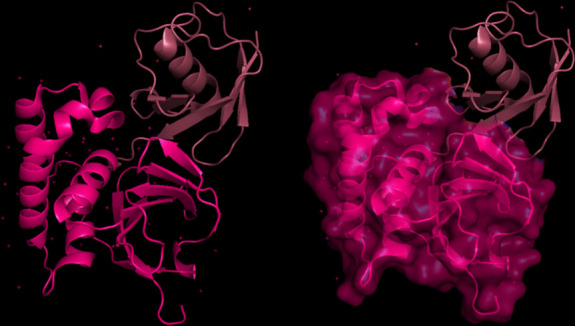
Chain (**a**) (hot pink) of the 3ZNH structure was visualized, corresponding to residues 1–183.

**Fig. 23:**
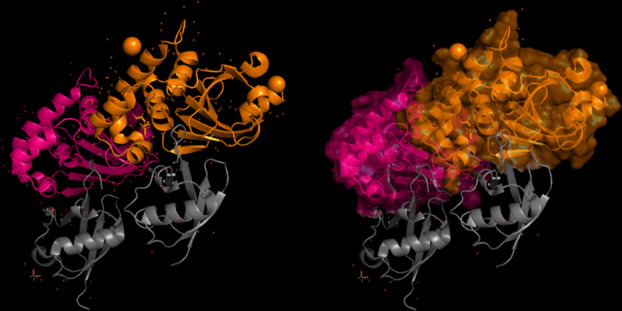
Chains (**a**) (hot pink) and (**c**) (orange) of the 5V5G structure were visualized, corresponding to residues 1–183.

**Fig. 24:**
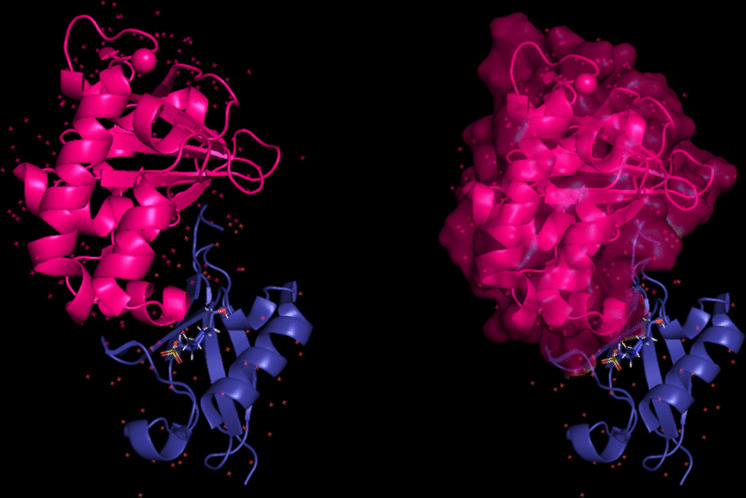
Chain (**a**) (hot pink) of the 5V5H structure was visualized, corresponding to residues 1–169.

**Fig. 25:**
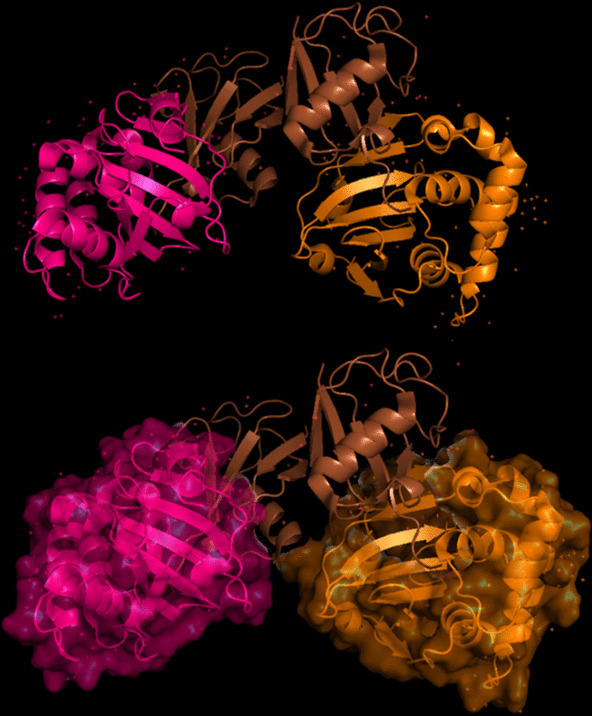
Chains (**a**) (hot pink) and (**c**) (orange) of the 5V5I structure were visualized, corresponding to residues 1–169.

**Fig. 26:**
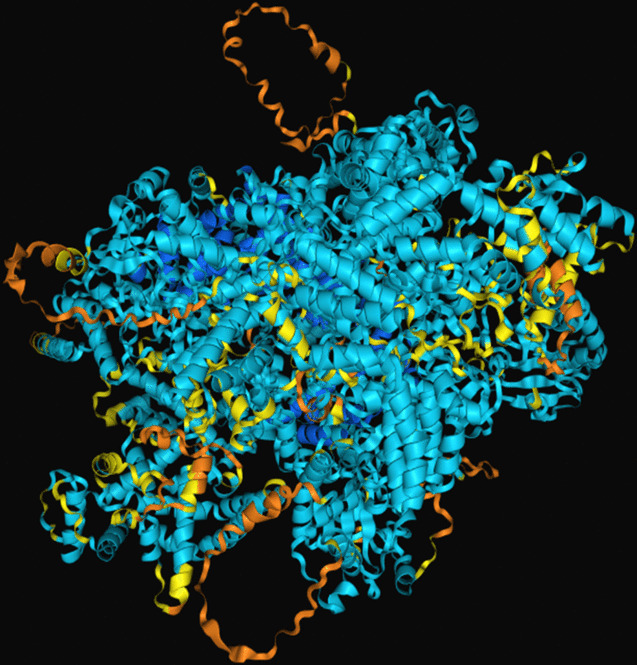
The most reliable 3D model of the Q6TQR6 protein obtained from AlphaFold DB.

The closest 3D structural model of the Q6TQR6 protein was generated using the AlphaFold DB (Fig. 26). The predicted ipTM = –pTM value of 0.75 exceeds the reliability threshold of 0.5, indicating a highly confident structural prediction.

## Discussion

CCHFV remains one of the most lethal tick-borne pathogens affecting humans, and despite decades of study, no approved vaccine is currently available. Therefore, identifying potential immunogenic targets through bioinformatics analyses is essential to guide future vaccine development. In this study, the physicochemical, antigenic, and toxicity properties of two CCHFV proteins, Q8JSZ3 and Q6TQR6, were analyzed *in silico* to evaluate their suitability as vaccine candidates.

The findings demonstrated that both proteins exhibited non-toxic and non-allergenic properties while maintaining antigenicity above the threshold value commonly accepted for vaccine consideration. The predicted antigenicity value of Q8JSZ3 was higher than that of Q6TQR6, suggesting stronger immunogenic potential. However, the structural and physicochemical properties of these proteins provide additional insights that are critical for assessing their biological relevance as vaccine targets.

One of the most notable distinctions between the two proteins is the number of predicted transmembrane helices. Q8JSZ3 was predicted to contain five transmembrane regions, whereas Q6TQR6 contained none. The presence of multiple transmembrane helices in Q8JSZ3 suggests that most of the protein is anchored within the viral membrane, potentially limiting accessibility to immune recognition. Nevertheless, exposed extracellular loops or surface domains within transmembrane proteins often contain key antigenic epitopes that can induce neutralizing antibody responses. Previous studies have shown that CCHFV glycoproteins include surface-exposed epitopes responsible for initiating protective immune responses in infected hosts [22]. Thus, the transmembrane nature of Q8JSZ3 should not automatically disqualify it as a vaccine candidate but highlights the need for focused epitope mapping of its extracellular regions in future studies.

Conversely, Q6TQR6 lacks transmembrane helices, indicating that it functions as an internal viral protein, most likely within the replication machinery of the virus. Internal proteins such as polymerases generally contribute to cellular immune responses rather than humoral responses by providing conserved cytotoxic T-lymphocyte (CTL) epitopes. Such epitopes are critical for inducing T-cell-mediated immunity, which plays an essential role in viral clearance and long-term protection. Indeed, previous immunoinformatics analyses of CCHFV polymerase proteins identified multiple conserved CTL epitopes suitable for incorporation into multi-epitope or peptide-based vaccine formulations [23]. Therefore, Q6TQR6 may complement surface antigens like Q8JSZ3 within a multivalent or subunit vaccine design.

Both proteins were predicted to be unstable according to their instability index values, which were higher than 40. Instability may pose challenges for protein expression and purification during recombinant production. However, several strategies could overcome this limitation. Codon optimization for the selected expression host, incorporation of stabilizing mutations, and fusion with carrier or chaperone molecules can enhance folding efficiency and overall stability. These optimization steps are commonly employed in vaccine construct design and can convert an otherwise unstable antigen into a viable immunogen for experimental testing.

Comparing the predicted antigenic features of both proteins with known immunogenic epitopes and vaccine design strategies provides biological justification for their consideration as vaccine targets. Previous studies on CCHFV glycoproteins have identified highly conserved, surface-exposed epitopes capable of eliciting strong neutralizing antibody responses [22]. In contrast, internal viral proteins like polymerase and nucleoprotein have been proposed as T-cell vaccine components due to their conserved nature and lower mutation rates, which may provide cross-strain immunity [23, 24]. Therefore, combining epitopes from both Q8JSZ3 and Q6TQR6 could form a rational strategy for a broad-spectrum, multi-epitope CCHFV vaccine design that targets both humoral and cellular immunity.

It is also important to emphasize that all findings presented here are based on computational predictions. Although bioinformatics analyses provide valuable preliminary information about antigenicity, allergenicity, and toxicity, they cannot experimentally confirm immunogenicity or safety. Experimental validation—including recombinant expression, antigen–antibody interaction assays, and *in vivo* immunogenicity testing—will be essential to verify these results.

Taken together, the computational analyses indicate that **Q8JSZ3** and **Q6TQR6** possess favorable antigenic, non-toxic, and non-allergenic characteristics, suggesting their potential as vaccine targets that merit further *in vitro* and *in vivo* validation. Notably, **Q8JSZ3** demonstrates higher predicted antigenicity and a membrane-associated profile, features consistent with previously characterized immunogenic glycoprotein regions of CCHFV. Conversely, epitopes derived from **Q6TQR6** could be incorporated into future multi-epitope vaccine designs to enhance T-cell mediated responses and broaden protective coverage. These results highlight the significance of integrating *in-silico* predictions with experimental research to advance rational vaccine development against CCHFV.

## Conclusions

In this study, Q8JSZ3 and Q6TQR6 proteins of CCHFV, a pathogen with high mortality rates and significant public health impact, were analyzed using bioinformatics approaches. Based on the comparative evaluation of physicochemical, antigenic, and toxicity profiles, Q8JSZ3 demonstrated higher antigenicity than Q6TQR6,suggesting its greater potential as a vaccine candidate. Given its non-toxic and non-allergenic nature, Q8JSZ3 could serve as a promising target for future *in vitro* and *in vivo* studies aiming to develop effective and safe vaccine formulations against CCHFV.

## Data Availability

In silico data is presented in the manuscript. There is no further data to present.
